# Effects of neuromuscular training on pain intensity and self-reported functionality for patellofemoral pain syndrome in runners: study protocol for a randomized controlled clinical trial

**DOI:** 10.1186/s13063-019-3503-4

**Published:** 2019-07-09

**Authors:** Haoyu Hu, Yili Zheng, Xiaochen Liu, Di Gong, Changcheng Chen, Yizu Wang, Mengsi Peng, Bao Wu, Juan Wang, Ge Song, Juan Zhang, Jiabao Guo, Yulin Dong, Xueqiang Wang

**Affiliations:** 1 Department of Sport Rehabilitation, Shanghai University of Sport, 399 Changhai RD, Shanghai, China; 2 Department of Rehabilitation Medicine, Shanghai Shangti Orthopedics Hospital, Shanghai, 200438 China

**Keywords:** Neuromuscular training exercise, Patellofemoral pain syndrome, Randomized controlled trial

## Abstract

**Background:**

Patellofemoral pain syndrome (PFPS) is common and affects approximately 15% of individuals at different ages and activity levels. As a non-surgical intervention, physiotherapy is widely used to treat PFPS. Neuromuscular training exercise is one of the most effective methods for decreasing musculoskeletal pain and improving knee function. However, the effectiveness of neuromuscular training exercise for treating PFPS is not without argument. The purpose of this study is to evaluate the effect of neuromuscular training exercise on patellofemoral pain and whether the neuromuscular training exercise have more advantage effects than taping and health education.

**Methods:**

We will operate a prospective, single-blind, randomized controlled trial of 60 patients with patellofemoral pain. Individuals will be indiscriminately assigned to two intervention groups and a health education group. The neuromuscular training exercise which includes the muscle strength training, balance training and knee joint proprioception training, and taping group will use “Y” and “I” type taping on the participants three times a week for three months. The health education group will be given education lectures once each week and which last for three months. The primary outcome measures include the adverse events, visual analog scale for pain, and Anterior Knee Pain Scale Index, which is a knee function self-report questionnaire to evaluate the function of the knee especially for PFPS patients. The secondary outcome measures are the muscle strength and endurance of knee joint flexion and extensor muscles, knee joint proprioception, muscle thickness of the quadriceps femoris, knee function ability, and quality of life. We will manage the intention-to-treat analysis for individuals who will withdraw from this study.

**Discussion:**

According to previous studies, neuromuscular training exercise and the taping method are effective treatment for PFPS patients. In this study, we will perform a neuromuscular training exercise for patients with PFPS. We believe that this study may prove the effectiveness of neuromuscular training exercise in treating PFPS.

**Trial registration:**

Chinese Clinical Trial Registry, ChiCTR1800014995. Registered on 27 February 2018.

## Background

Patellofemoral pain syndrome (PFPS), also known as anterior knee pain, runner’s knee, and chondromalacia patellae, is one of the most common knee problems among runners and individuals after injury or surgery around the knee [1]. In the field of sports medicine, PFPS accounts for 25% of all present running injuries [2]. The prevalence of PFPS is in the range of 11–17% [3, 4]. PFPS is also particularly common in women [2]. With its high prevalence, PFPS may cause a huge economic burden due to medical spending and lost remuneration as the syndrome may last for 20 years [1, 5]. If we want to effectively treat PFPS patients, we should first know the cause of the syndrome.

PFPS is multifactorial and the causes are unclear [6]. However, as the biomechanical structure of the knee joint has strong correlations with the hip and ankle joints, the causes of PFPS are complicated [7, 8]. According to the previous study, the main reasons may be as follows: the different activation rates of medial femoral muscle and lateral femoral muscle, especially at the end of knee extension; abnormal Q-angle, which is more common in women [9–11]; weakness of the gluteal muscle, especially in the middle gluteal muscle, which leads to decreased abduction and rotation of the hip joint during exercise [2, 12, 13]; and femoral anteversion or tibial torsion [1, 11].

The current treatment for PFPS now focuses on conservative methods, among which physical therapy plays an important role [2, 14]. Physical therapy is widely used to manage the dysfunction and lessen the pain experienced by PFPS patients. It advocates exercise training for decreasing the pain and improving the knee function of patients [11]. Neuromuscular training exercise is a common treatment method in physical therapy; it includes muscle strength training, balance training, and proprioception training [13, 15, 16]. Taping is another effective way to treat PFPS patients [17–19]. Currently, most scholars use neuromuscular training exercise and taping as a combined treatment for PFPS patients [18].

Although neuromuscular training exercise and taping are recommended treatments for PFPS patients, their effectiveness still remains disputed. On the one hand, several studies proved that neuromuscular training exercise and taping have positive effects in the treatment of PFPS, especially in improving muscle strength and knee joint proprioception sense ability [12, 16]. The scholar Hande Guney reported that quadriceps strength and joint position sense are strongly correlated with pain and knee function in PFPS patients [20]. On the other hand, several studies proved that these treatments may not be efficacious in improving the muscle strength or joint position sense ability of patients with PFPS [21, 22] and joint position sense may not have any difference between PFPS patients and normal individuals [23].

The objective of the present study is to determine whether neuromuscular exercise training and taping are effective in improving knee function and lessening the pain in PFPS patients. Thus, we will design a single-blind randomized controlled trial (RCT). Compared with other studies, we will ask individuals to follow several muscle strength-training regimens, including quadriceps femoris and gluteus strength training, especially the medial vastus muscle. Moreover, we will use a balance pad to train the balance ability and CON-TREX multijoint isokinetic test and training machine to train the patients’ knee proprioception sense ability.

According to previous studies, health education is important among people who suffered from PFPS [24–26]. As a result, we set up a control group named “health education;” the control group will be used to compare with the other two groups. In addition, the health education program contents are as follows: it will give the participants publicizing and knowledge about how to protect the knee from injury while they are running or in the daily life as well as the concept of PFPS and self-management strategies on it.

### Hypothesis

1. The neuromuscular training exercise and taping both have a positive effect on the treatment of PFPS.
2. The neuromuscular training exercise has a higher advantage than taping and health education in treating PFPS patients.

## Methods

### Sample size estimation

According to the previous studies, the effect size of Kujala Patellofemoral Scale is in the range of 0.34–0.54. Pain intensity effect size is in the range of 0.45–0.56 [2]. In this study, we used G-Power Software: F-test, ANOVA: one-way repeated measures (version 3.1.9.2) with the following parameters: effect size = 0.3; test level = 0.05; test efficacy = 0.95; group numbers = 3, number of measurements = 3. The total sample size of this study should be a minimum of 60 participants in the three groups, or 20 participants in each group.

### Purpose of the study

This study will operate a RCT including neuromuscular training exercise and taping in patients with PFPS to determine the following:

- whether neuromuscular training exercise is effective for treating PFPS;
- whether neuromuscular training exercise has more advantageous effects than taping and health education for PFPS patients;
- the adverse effects of neuromuscular training exercise.

### Study design

**Fig. 1:**
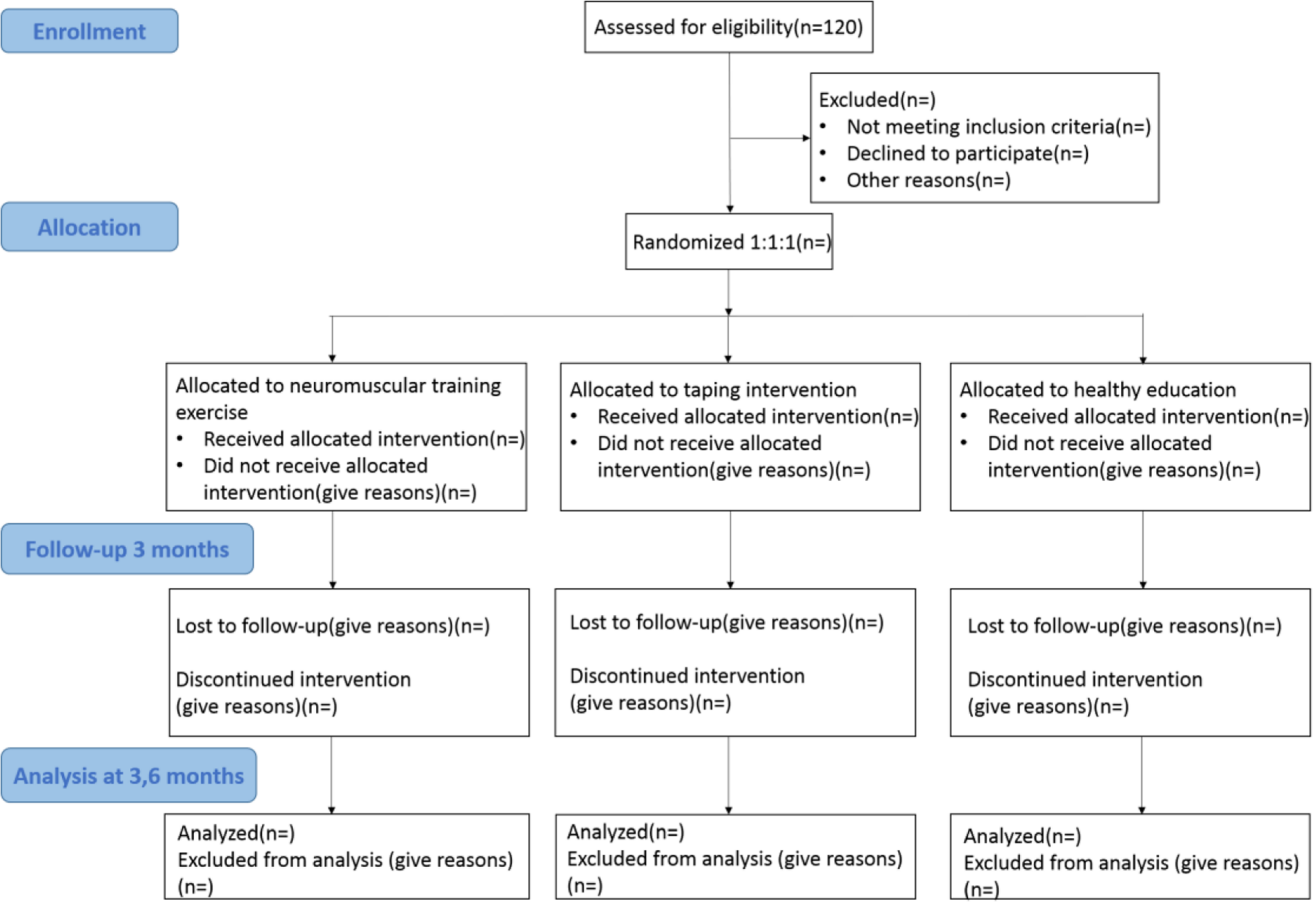
*Flow diagram* showing neuromuscular training exercise for PFPS patients

We will design a single-blind RCT to compare neuromuscular training exercise with taping and health education (Fig. 1). We will recruit and investigate a total of 120 patients with PFPS from Shanghai Shangti Orthopedic Hospital and Shanghai University of Sport, Shanghai City, China, and different running teams from Shanghai.

Before starting the study, all individuals will fill in a questionnaire including basic personal information, such as age, educational background, and injury history. The questionnaire will also include a section on pain intensity (visual analog scale [VAS]) and knee function (Anterior Knee Pain Scale [AKPS]). Before inclusion, informed consent will be obtained from all participants.

All individuals who conform to the study will be randomly assigned in a 1:1:1 ratio by a computer. Afterward, patients with PFPS will be distributed to the control group (health education), first intervention group, (neuromuscular training exercise), or the second intervention group (taping). The whole study will last for six months, which includes three months of intervention and another three months of follow-up period without intervention.

The assessments will be conducted before the intervention, at six weeks, at three months, and at six months.

### Participants

#### Inclusion and exclusion criteria

The participants should meet the following requirements: (1) aged 20–50 years; (2) running more than half a year and pain lasting longer than one month; (3) anterior or retropatellar knee pain from at least two of the following: prolonged sitting; climbing stairs; squatting; running; kneeling; and hopping/jumping; (4) anterior knee pain is not caused by trauma; (5) the patellofemoral joint extrusion test is positive. The exclusion criteria are as follows: (1) meniscus injury; (2) laxity of articular ligament; (3) pressure pain on the tibial tract, the goose foot tendon, and patellar ligament; (4) effusion of knee joint; (5) history of patellar dislocation; (6) severe cardiovascular or osteoporosis, progressive neurological deficits; (7) pregnancy women or lactating women; (8) VAS score (in the range of 1–10) > 8; and (9) VAS score (in the range of 1–10) < 3.

### Withdrawal criteria and management

PFPS patients will be asked or be allowed to quit the study if:

- they have such a demand;
- they are diagnosed with a severe disease, such as cardiovascular disease;
- they obtain a side effect because of treatment.

### Interventions

All the groups will receive basic information and intervention details individually before they enter the study. The individuals will be required to fill in forms to record the durations and times of intervention. The patients will need to keep their normal lifestyle and avoid taking any other rehabilitation treatments.

### Neuromuscular training exercise group

**Fig. 2:**
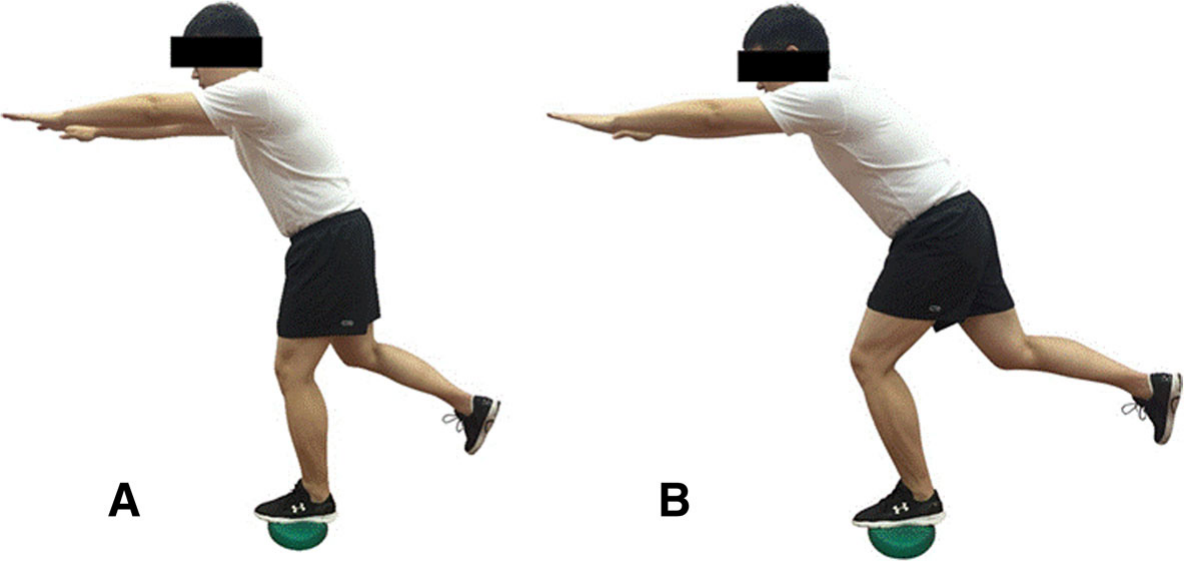
Balance training exercise: (**a**) knee flexion at 30°; (**b**) knee flexion at 60°

**Fig. 3:**
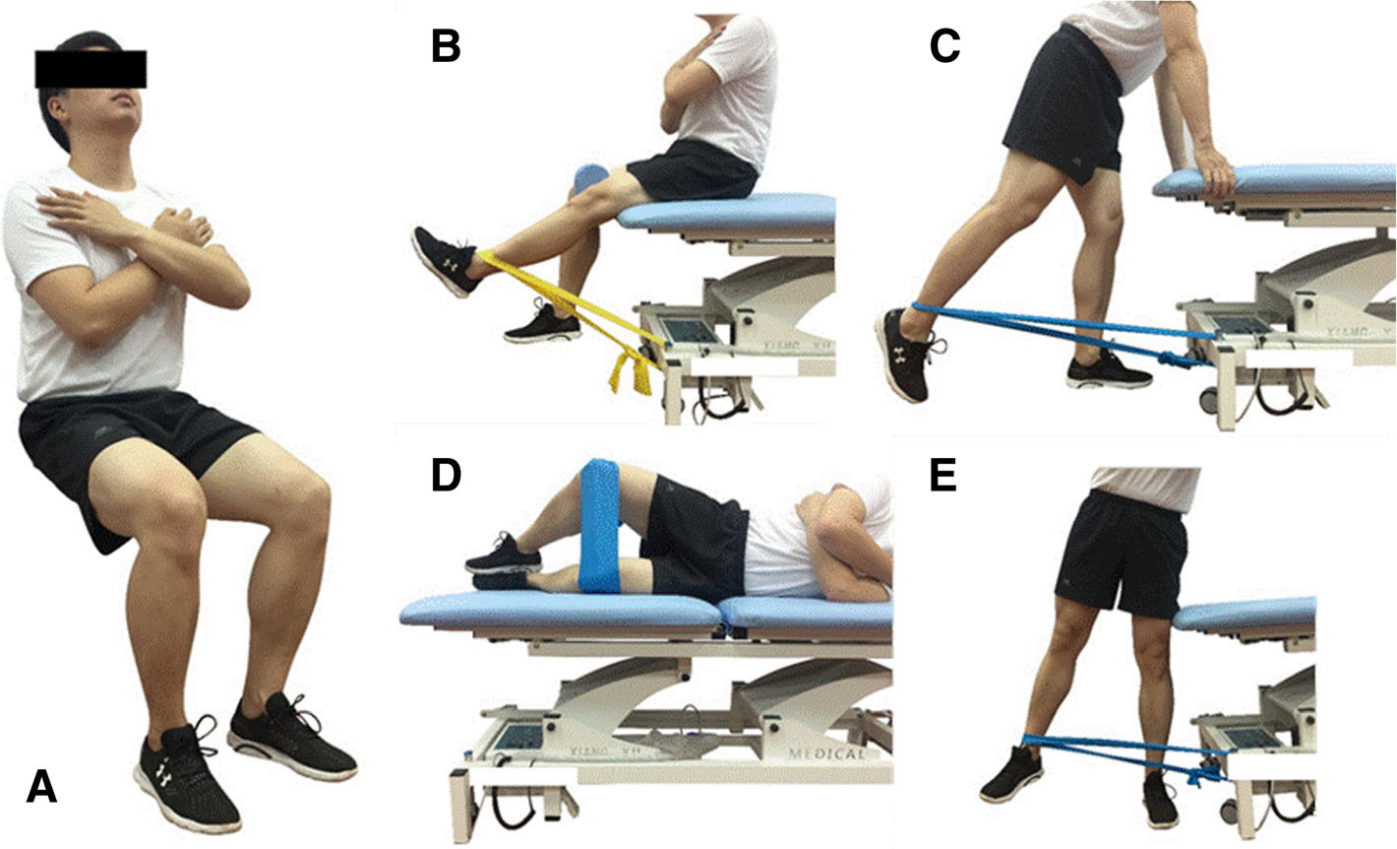
**a–e** Muscle strength training in five different exercises

**Fig. 4:**
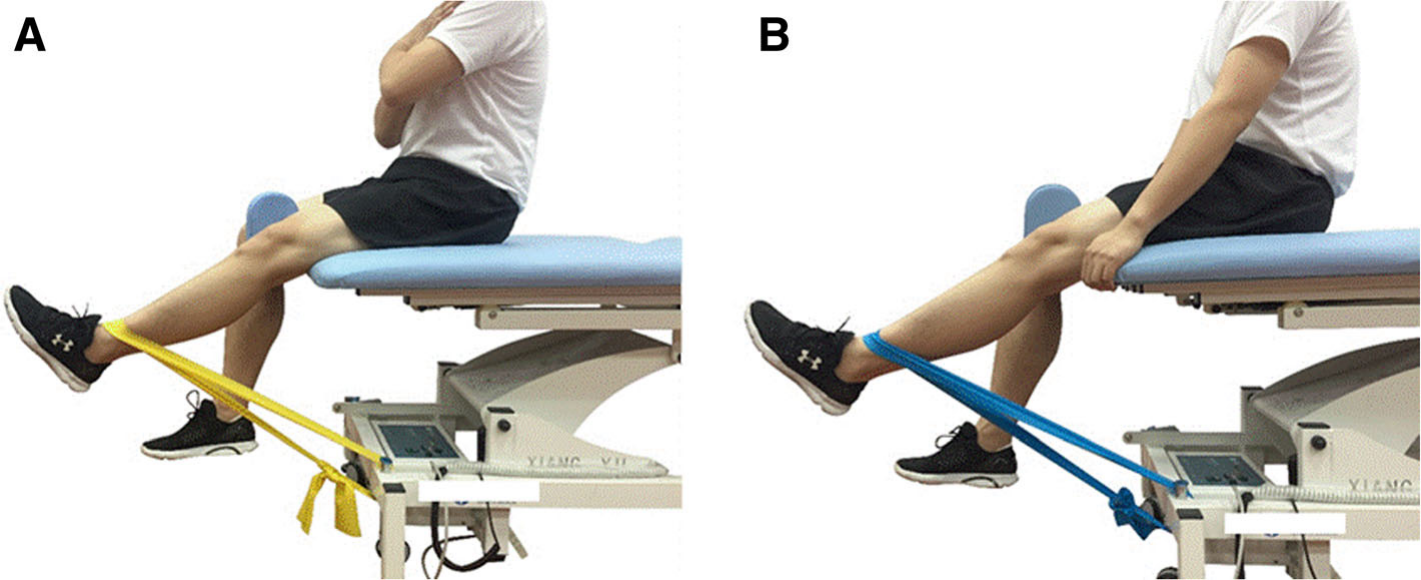
Different stretching force elastic bands: (**a**) low intensity; (**b**) high intensity

The participants will perform the neuromuscular training exercise, including the quadriceps femoris and gluteal muscle strength training, balance training (Fig. 2), and knee proprioceptive sense training. The muscle strength training includes five different exercise training movements (Fig. 3). All individuals should come to the clinical center three times a week. The whole exercise routine has three parts: warm up for 5 min; neuromuscular training exercise for 40 min; and muscle stretching for 5 min. The physical therapist will give the participants a guideline for each type of movement. The training exercise regimen will be different according to the period and participants’ body condition. We will normally increase the training intensity every two weeks by expanding the training time and convert to a more difficult exercise (Figs. 2 and 4), such as deep squats with different angles and using different stretching force elastic bands (blue: high intensity, yellow: low intensity).

### Taping group

**Fig. 5:**
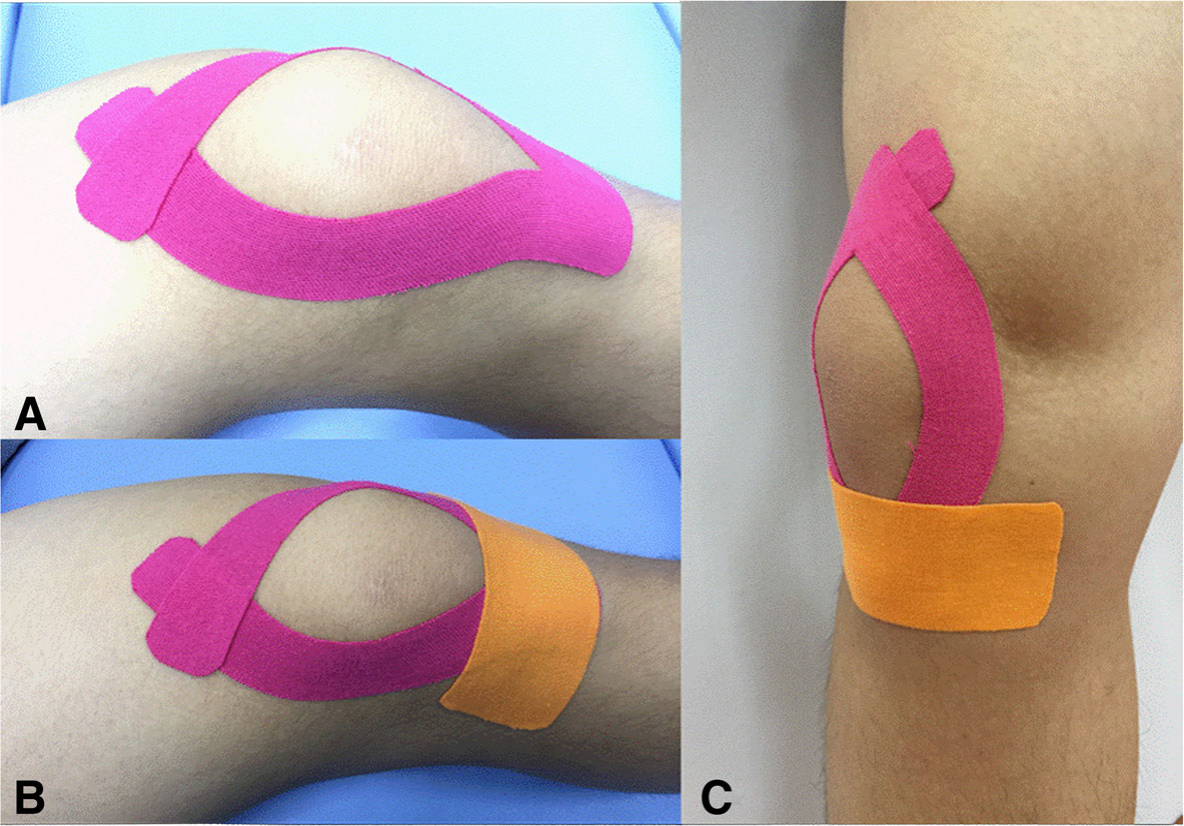
**a** Y-shaped taping. **b**, **c** Y- and I-shaped taping

A certified physical therapist will tape participants with a Y-shaped tape (Fig. 5) from the lower edge of the patella to the middle segment of the quadriceps femoris and an I-shaped tape according to Kenzo Kase’s Kinesio taping manual. The tape therapy will last for three months and all individuals should come to the clinical center three times a week.

### Health education group

The participants in this group will attend sessions on protecting the knee from injury while they are running or in the daily life for once a week. In addition, we will use the Internet to broadcast some articles about the PFPS concept and how to deal with it. The health education sessions will also last for three months.

### Outcome measures

#### Primary outcome measures

1. We will use the VAS scores to evaluate pain intensity. Normally, the VAS comprises a horizontal line with a length of 100 mm and a pain scale of 0–10, where the score “0” means no pain and “10” unbearable pain. The pain intensity is described by the patient who points to a number from the line [27].
2. The knee function self-report questionnaire will be administered. The results will be evaluated using the AKPS. The Chinese translated version of the AKPS is a reliable and valid questionnaire for patients with PFPS [28–30]. It is a 13-item knee-specific self-report questionnaire used to record a patient’s reaction to six different activities, which may have specific connections with PFPS syndrome. The AKPS is used to record the duration of symptoms and which limb(s) is/are affected. Its full score is 100 and lower scores mean significant pain or severe knee function.
3. The adverse events, which have correlations with neuromuscular training exercise and taping, will be recorded.

#### Secondary outcome measures

1. The quadriceps femoris strength and endurance indexes will be tested using the CON-TREX multijoint isokinetic test and training machine (CMV AG, Dübendorf, Switzerland). The maximum concentric contractions for knee flexors and extensors of individuals will be recorded at different angular velocities of 60°/s, 120°/s, and 180°/s. The muscle endurance index will be obtained according to the ratio of the work done during the last five contractions over the first five contractions. The parameters will be recorded as peak torque (Newton-meters), peak torque/body weight, power output (power) in watts, and work (work) in joules [20].
2. The knee joint proprioception sense ability will also be evaluated using the CON-TREX multijoint isokinetic test and training machine. The individuals will be asked to move to a random reference position, hold this position for 3 s, and then repeat from the neutral position to the reference position. Next, the participants will find the nearest position and will keep this position so that the assessor can measure the position and angle. The knee joint proprioception test will be repeated three times and the final results will be averaged. A high absolute error means poor proprioceptive sense [31].
3. The muscle thickness of the quadriceps femoris will be determined. We will use the portable musculoskeletal ultrasonic instrument (M7 Super Series, Mindray, USA) to evaluate four different muscles: rectus femoris, vastus intermedius, vastus lateralis, and vastus medialis. The methods we will follow are based on the studies by Giles [32, 33].
4. We will use three tests to evaluate the knee joint function: anteromedial lunge test; step-down test (left and right); and balance-and-reach test. These tests have good reliability and can better reflect the function of knee joint [34].
5. Quality of life will be evaluated using the Short Form 36 (SF-36) health survey. The survey is divided into eight different parts: vitality; physical functioning; bodily pain; general health perceptions; physical role functioning; emotional role functioning; social role functioning; and mental health (the psychometric properties). The score of SF-36 is from 0 (worst) to 100 (best) [35].

### Statistical analysis

Statistical analyses will be carried out using SPSS 22.0 and Microsoft Excel 2012 software. Data will be represented as mean ± standard deviation. We will use two-way repeated measures analysis of variance (group × time) to compare the effect of neuromuscular training exercise group with the taping and control groups, including the primary and secondary outcomes. The intention-to-treat analysis will be conducted if individuals are lost to follow-up. The t-test will be carried out to compare the changes in measures within groups. Statistical significance will be considered at *P* value < 0.05.

## Discussion

There is no consistency standard for the treatment of PFPS. According to a previous study [36], neuromuscular training exercise should be an effective treatment for PFPS patients. In this study, we will carry out an RCT of neuromuscular training exercise and taping for patients with PFPS. We believe that our study will provide evidence of the effectiveness of neuromuscular training exercise in treating PFPS. Compared with health education and taping, neuromuscular training exercise could provide more beneficial effects on alleviating the pain, ameliorating knee function, and improving the PFPS patients’ quality of life.

### Strengths and limitations

First, most previous studies focused on decreasing PFPS pain intensity. However, in this study, we also make assessment on the knee joint function. In addition, we will use several knee function movements for assessment and to determine the effect of treatment. Second, we will evaluate knee proprioception in three groups. We want to determine whether the intervention group will have better knee proprioception sense compared with the other two groups. According to another study, knee proprioception plays an important role in improving knee function [18]. Third, we will set up three groups, namely, neuromuscular training exercise, taping, and health education groups, which will make the research more rigorous and comprehensive.

Despite its strength, this study has some potential limitations. The exercise training routines may not be suitable for all participants, especially those with high pain level.

In this study, we want to determine the effectiveness of neuromuscular training exercise for PFPS. We also want to estimate which method will have more beneficial effects for PFPS patients. We hope that our study will benefit patients, researchers, and policy makers with an interest in the treatment of PFPS.

### Trial status

The study registration number is ChiCTR1800014995 and the recruitment still in progress. The recruitment may be finished in October 2018.

## Data Availability

The data and the relevant results in this study will be shared through the academic conferences and the scientific papers.

## Abbreviations

AKPS: Anterior Knee Pain Scale
PFPS: Patellofemoral pain syndrome
RCT: Randomized controlled trial
SD: Standard deviation
SF-36: Short form 36
VAS: Visual analog scales
